# Elder abuse, resilience, and health-related quality of life: evidence from a cross-sectional study among older adults in Sanandaj, Iran

**Published:** 2026-01

**Authors:** Roya Asadi, Serveh Parang, Sargol Shafiee, Kamyar Mansori, Leila Hashemi Nasab, Jamal Seidi

**Affiliations:** ^*a*^ Student Research Committee, Kurdistan University of Medical Sciences, Sanandaj, Iran.; ^*b*^ Department of Midwifery, Clinical Care Research Center, Research Institute for Health Development, Kurdistan University of Medical Sciences, Sanandaj, Iran.; ^*c*^ Department of Epidemiology and Biostatistics, School of Medicine, Zanjan University of Medical Sciences, Zanjan, Iran.; ^*d*^ Clinical Care Research Center, Research Institute for Health Development, Kurdistan University of Medical Sciences, Sanandaj, Iran.

**Keywords:** Elder adult, Older Adults, Elderly Abuse, Resilience, Quality of Life

## Abstract

**Background:**

Elder abuse constitutes a significant violation of human rights and is linked to various harmful psychological and physical consequences. This research aimed to investigate these effects by assessing the prevalence of elder abuse and its relationship with resilience and health-related quality of life among older adults who attended the comprehensive health centers in Sanandaj, Iran, in 2023.

**Methods:**

This study employed a cross-sectional design that involved 200 elderly individuals who sought services at comprehensive health centers in Sanandaj city, located in the Kurdistan province of Iran, in 2023. Participants were chosen through a two-stage process. The data collection instruments included a form for recording demographic information, a questionnaire on elder abuse, and the CD-RISC measure. Data were analyzed using univariate and multivariate logistic regression, along with the Pearson Correlation test in STATA12 software, setting the significance level p-value ≤ 0.05.

**Results:**

The findings revealed that 57.5% of the participants had encountered some form of abuse, with psychological abuse being the most prevalent at 50%. The examination illustrated a significant relationship between the extent of elder abuse and the average mental health scores (p=0.001), resilience (p=0.001), living situation (p=0.05), and Educational level (p=0.05). Furthermore, there was a statistically significant relationship between resilience scores and mental health (r=0.572, p less than 0.001).

**Conclusions:**

In conclusion, the research indicated that a majority of elderly individuals had faced abuse, with higher occurrences among those who were illiterate and resided in rural settings. The findings also suggested that older adults with greater resilience scores experienced lower levels of elder abuse and reported better mental health.

## Introduction

According to the World Health Organization, individuals who are 60 years old and above fall into the category of elderly people.^1^This age demographic is growing swiftly across the globe,^2^ and projections suggest that the population of older adults will approach two billion by the year 2050.^1^ In Iran, demographic projections show that by 2032, individuals in this age group will comprise about 25–30% of the national population.^3^

Growing older is a phase of human existence that brings about changes both physically and mentally, potentially resulting in various health-related and psychological challenges.^4^ A study by Mohaqeqi Kamal et al. ^5^ showed that 79.8% of Iranian elder adult individuals suffer from chronic diseases, indicating a poor health status among the elder population. In Khalili et al.'s^6^ study, 57.3% of the elder adult in Sari did not have good health status. Elder adult health is an important issue for individuals and society, as it allows them to experience the best possible quality of life by maintaining independence, active participation in social and economic affairs, reducing health and medical expenses, maintaining mental health, and increasing life expectancy.^7^The wellbeing of senior individuals is influenced by numerous factors, including their socioeconomic circumstances, psychological traits, and the accessibility of healthcare services.^8,9^ In addition, experiencing elder abuse is another influential factor that can significantly affect their overall well-being.^10^

Elder mistreatment refers to any act or failure to act—whether a one-time event or a recurring issue—within a relationship that is expected to be trusting, which results in harm or distress to an elder adult. This abuse can manifest in different ways, including physical, sexual, psychological or emotional mistreatment, financial exploitation, and neglect.^11^ Abuse is divided into two general categories based on the place of occurrence and the perpetrator: domestic and organizational. Domestic abuse refers to violence inflicted by someone familiar to the elder adult within their home, whereas institutional elder abuse takes place in facilities such as nursing homes or long-term care environments, perpetrated by staff or caregivers.^12^

The global rate of elder abuse is on the rise.^13^ A systematic review conducted in 2017 across 28 countries found that around one in six individuals aged 60 and over had experienced some form of violence or mistreatment.^14^ Such experiences are considered major sources of life stress for older adults,^15^ often leading to complications such as a decrease in self-confidence, hopelessness, indifference, mental and psychological problems, disability,^16^ depression, a decrease in general health^17^ and impairment in the social functioning of the elder people.^18^

The occurrence of physical, mental or social disorders, in the face of severe stress, requires a biological background in a person, while most people are susceptible to some diseases and disorders, many of these problems never arise. A factor that protects people from the adverse consequences of stress is the difference in the ability to cope with stress and resilience.^19^ Resilience, as a factor of internal resistance, helps individuals regulate stress by increasing their problem-solving ability and motivation to deal with problems. It is a crucial factor in reducing the consequences of elder abuse.^20^ Cuevas et al. ^21^ showed in their study that the likelihood of exposure to elder abuse is lower among people who are more resilient, and their health improves with a decrease in elder abuse.

As the older population continues to expand and societal changes brought on by urbanization weaken traditional values, there is an increasing need to prioritize the health of older adults and consider factors influencing it, such as elder abuse and resilienc.^22^ Differences in reported prevalence among various studies indicate that cultural, economic, and policy-related contexts significantly impact both the incidence of elder abuse and the overall well-being of older adults in diverse environments. Based on these insights, this study aimed to evaluate the prevalence of elder abuse and its relationship with resilience and health-related quality of life among older adults utilizing services at the Sanandaj Comprehensive Health Centers. Based on the study objectives, the following hypotheses were proposed:

1. There is a significant relationship between elder abuse and health-related quality of life among older adults.

2. There is a significant relationship between elder abuse and resilience among older adults.

3. There is a significant relationship between resilience and health-related quality of life among older.

## Methods

This descriptive-analytical with cross-sectional design study was conducted in 2023 and included all eligible older adults who attended the comprehensive health centers in Sanandaj, situated in Kurdistan Province, Iran. The criteria for inclusion were being 60 years or older, having the ability to communicate effectively, no impairments in hearing or vision, and no cognitive impairment (with a score of 6 or higher on the cognitive impairment test) and no confirmed psychological disorders. Questionnaires that were incomplete were excluded from the analysis. The sample size was determined using a Cochran's formula,23 with a confidence interval of 99%, z1-a/2=2.576, d= 0.1 and P=0/5.

$$ n=\frac{Z_{1-\alpha/2}^{2}\,p(1-p)}{d^{2}} $$

Based on an effect size of 25%, the final sample size was estimated to be 200 individuals, were selected in two stages; initially a list of comprehensive health centers in the four districts of Sanandaj city was compiled. Subsequently, three centers were randomly chosen in each district. In the second stage, participants were selected from eligible elderly attending these centers using a consecutive convenience sampling method.

Prior to conducting the study, necessary approvals were obtained from the ethics committee (IR.MUK.REC.1401.161). First, the cognitive status of elderly individuals attending comprehensive health centers was evaluated using a short form of the cognitive test. Once eligible elderly participants were identified, the study’s purpose was explained to them, highlighting that all information would remain confidential. After that, written informed consent was gathered. Participants then completed a demographic information form, the Elderly Abuse Questionnaire (EAQ), the Persian version of SF-12, and the Connor-Davidson Resilience Scale (CD-RISC) which has been psychometrically measured in studies by Montazeri et al.^24^ and Rezaei Pandari et al.^25^ These forms were filled out either independently by literate participants or through interviews for those who were illiterate, all conducted in a private environment.

The demographic profile registration form consisted of 11 elements: age, gender, educational level (having formal education), marital status, residence location, housing situation, number of children, employment status, monthly household income, insurance coverage and smoking habit. The validity of this form was established using content validity method. The Elderly Abuse Questionnaire (EAQ) evaluated occurrences of elder abuse within the previous year using 13 items, categorized into three groups: physical abuse (5 items), psychological abuse (4 items), and financial abuse (4 items). Responses were documented as "yes” or "no,” with a positive answer to any single item considered indicative of elder abuse. Rashidi Fakari and colleagues^26^ assessed the psychometric characteristics of this instrument specifically for the Iranian population, finding Cronbach's alpha values of 0.8 for the overall scale and 0.82, 0.7, and 0.72 for the subscales of physical, psychological, and financial abuse, respectively. The SF-12 questionnaire consist of physical (6 items) and mental (6 items) dimensions of health with mixed response formats, including 3, 5 and 6 point Likert-type scale. The total scores ranges from 12-48; higher scores reflect better health-related quality of life. The 10-item Connor-Davidson Resilience Scale (CD-RISC) measures an individual's ability to cope with stress. Participants answered using a 5-point Likert scale, from 0 ("Not true at all”) to 4 ("True nearly all the time”), producing total scores between 0 and 40, where higher scores indicate increased resilience.

Data analysis was conducted utilizing both univariate and multivariate logistic regression, along with the Pearson Correlation test, using the STATA12 software. A significance level of 0.05 was used for this analysis.

## Results

The mean age of the participants was 69.8 years with a standard deviation of 8.4, with an average number of children 4.8±2.07. The majority of participants were women (56%), literate (55.5%), married (71.5%), urban residents (93%), living independently (77%), unemployed (45.5 %), had monthly incomes equal to their expenses (41.5%), had insurance coverage (86%), and were non-smokers (85.5%) (Table 1). In this study, 57.5% of participants reported being abused, with rates of physical, economic, and mental elder abuse at 16%, 35.5%, and 50% respectively. The average scores on the SF-12 questionnaire and resilience scales were 30.35±3.68 and 23.98±6.4, respectively. The mental health score (16.74±3.2) of the elderly was higher than their physical health score (13.61±1.1).

**Table 1:** Factors associated with elder abuse based on univariate regression model

Elder Abuse			Yes	No	B	OR	CI	P-Value
Variables			Mean ± SD	Mean ±SD				
Age			70.7±8.9	68.7±6.9	0.03	1.03	0.99-1.07	0.09 *
Number of children			4.9±2.11	4.6±2.02	0.07	1.07	0.93-1.23	0.32
mental Health			14.87±2.61	19.3±2.33	-0.67	0.51	0.43-0.61	0.001*
Physical Health			13.58±1.08	13.65±1.24	-0.05	0.95	0.75-1.21	0.69
Resilience			20.03±4.67	29.32±4.12	-0.575	0.56	0.48-0.66	*0.001
Variables	Frequency	N (%)	N (%)	Total	B	OR	CI	P-Value
**Sex**	**Female **	65(56.5)	47(55.3)	112(56)	**Constant**			
	**Male**	50(43.5)	38(44.7)	88(44)	-0.05	0.95	0.54-1.67	0.86
**educational level**	**Formal education**	72(62.6)	39(45.9)	111(55.5)	**Constant**			
	**No Formal education**	43(37.4)	46(54.1)	89(44.5)	-0.681	0.506	0.29-0.89	*0.019
**marital status**	**Single**	1(0.9)	2(2.4)	3(1.5)	**Constant**			
	**married**	75(65.2)	68(80)	143(71.5)	-1.65	0.19	0.02-2.28	*0.19
	**widow or divorced **	39(33.8)	15(17.6)	54(27)	0.86	0.42	0.22-0.84	*0.01
**place of residence**	**Urban **	102(88.7)	84(98.8)	186(93)	**Constant**			
	**Village**	13(11.3)	1(1.2)	14(7)	2.37	10.7	1.37-83.5	*0.02
**Housing Status**	**Independent **	84(73)	70(82.4)	154(77)	**Constant**			
	**Along with children or other relatives**	31(27)	15(32.6)	46(23)	0.26	1.3	0.61-2.77	0.49
**Employment status**	**Unemployed**	63(50.4)	28(37.3)	91(45.5)	**Constant**			
	**Retired or on pensioner**	58(46.4)	30(40)	88(44)	0.33	1.39	0.76-2.54	0.29
	**employed**	4(3.2)	17(22.7)	21(10.5)	-1.78	0.17	0.05-0.54	*0.003
**Monthly household income**	**No independent income**	20(16)	10(11.8)	30(15)	1.1	3	0.69-13.2	*.014
	**Less than expenses**	43(34.4)	34(40)	77(38.5)	0.64	1.9	0.49-7.3	0.35
	**Equal to expenses**	48(38.4)	35(41.2)	83(41.5)	0.72	2.1	0.54-7.8	0.29
	**More than expenses**	14(11.2)	6(7)	10(5)	**Constant**			
**Insurance coverage**	**yes**	99(86.1)	73(85.9)	172(86)	**Constant**			
	**No **	16(13.9)	12(14.1)	28(14)	-0.02	0.98	0.44-2.2	0.96
**Smoking habits**	**yes**	16(13.9)	13(15.3)	29(14.5)	0.11	1.14	0.2-2.47	0.78
	**No**	99(86.1)	72(84.7)	171(85.5)	**Constant**			

Multivariate logistic regression revealed that Place of residence and educational level showed borderline significant relationship with elder abuse (OR=0.068, p=0.05; OR=3.62, p= 0.05, respectively), whereas mental health (OR = 0.54, p = 0.001) and resilience (OR=0.65, p = 0.001) were significantly associated with elder abuse (Table 2 ). The average scores for resilience, SF-12, and its physical and psychological aspects were 23.98±6.4 (on a scale from 0 to 40), 3.68±3.35 (on a scale from 12 to 48), 13.61±1.15, and 16.74±3.32, respectively. A significant positive correlation exists between resilience (r=0.65, P=0.001) and the mental health of older adults (r=0.572, P=0.001) (Table 3).

**Table 2:** Predictors of elder abuse by multivariate logistic regression

C.I 95%	Sig.	OR	B	Variables	
0.97-2.94	0.06	1.4	0.531	age	
Constant				Formal education	educational level
1.001-13.01	0.05	3.62	1.07	no Formal education	
Constant				Urban	place of residence
0.004-1.11	0.05	0.068	-2.686	Rural	
Constant				Single	marital status
0.015-33.28	0.859	0.705	-0.349	married	
0.128-2.035	0.341	0.510	-0.672	widow or divorced	
Constant				Unemployed	Employment status
0.008-1.888	0.132	0.12	2.12	Retired or on pensioner	
0.527-7.827	0.303	0.709	2.031	employed	
Constant				No independent income	Monthly household income
0.009-4.38	0.303	0.194	-1.640	Less than expenses	
0.005-1.27	0.074	0.083	-2.485	Equal to expenses	
0.018-2.57	0.225	0.216	-1.532	more than expenses	
0.4-0.74	*0.001	0.54	-0.609	Mental Health	
0.56-0.77	*0.001	0.65	-0.431	Resilience	

**Table 3:** Correlation between resilience scale scores and dimensions of the SF-12 questionnaire

variables	physical health	mental health	Resilience
physical health	1	r=0.16	r=0.000
		p=0.025	p=0.997
mental health	r=0.16	1	r=0.572
	p=0.025		p=0.001
Resilience	r=0.000	r=0.572	1
	p=0.997	p=0.001	

Pearson Correlation test*

## Discussion

The present research sought to explore the connections between elder abuse, resilience, and health-related quality of life. The results indicated that a significant number of elderly participants (57.5%) had experienced some type of abuse. This result is consistent with Delpasand et al. ^27^, who reported a prevalence of 55% for elder abuse. In contrast, Askari et al. ^28^ found a higher prevalence of 78%**,** highlighting a discrepancy compared to the present study. The discrepancy in outcomes could be linked to the different evaluation instruments employed. In the present study, only economic, emotional, and physical dimensions were measured, while Askari et al. evaluated elder abuse across abandonment, caregiver neglect, mental, financial, physical, emotional abuse, denial of agency, and financial neglect dimensions.

As people age, their physical, mental, and psychological performance tends to decrease, making them more vulnerable to elder abuse.^29^ It is recommended that health care providers thoroughly screen elderly individuals for signs of abuse and provide appropriate care for them. However, intervening effectively to improve their situation may be challenging due to a lack of support systems for the elderly. As a result, the creation of support organizations for elderly by healthcare system administrators could also be advantageous in improving their well-being and overall quality of life.

The results of this study indicate that psychological abuse is the most prevalent form of mistreatment faced by older adults, aligning with the findings presented by Khalili et al. ^6^ in Sari city. Conversely, Darvishpour et al. ^30^ identified neglect in care as the predominant form of elder abuse. This difference may be explained by variations in the study populations: Darvishpour et al. focused on elderly individuals who were hospitalized and required more intensive care, whereas the current study involved elderly participants attending comprehensive health centers.

The high incidence of psychological abuse in Eastern societies may be linked to the cultural and social factors (e.g., changes in family roles, urbanization, and weakening of traditional values). Deep emotional connections between parents and children, as well as potentially unrealistic expectations they have of each other can also play a role. As societal conditions change, individuals may struggle to meet each other's needs effectively, leading to an increased likelihood of abuse being reported.^31^

The study's results indicated that elderly individuals living in rural and illiterate communities experienced higher rates of elder abuse. Additionally, Najafi Moghadam et al. ^32^ found a correlation between living in rural areas and an elevated risk of elder abuse. Elements such as social isolation, restricted access to support services, and financial reliance were identified as contributing factors to the increased rates of elder abuse in rural regions. To tackle this problem, creating support networks and peer groups within rural communities, along with improving cooperation between health and social organizations, could be beneficial in addressing elder abuse.

In the present study, the resilience of the elderly was found to be moderate. The findings of Khalili et al.'s ^6^ study also supported the results of this study, while Bahrami et al.'s ^33^ study reported a lower level of resilience among the elderly. The differences in the findings reported by various studies could be related to changes in physiological and environmental factors, personal experiences, cultural and economic circumstances, as well as social dynamics among the research communities.

High resilience reflects an individual’s capacity to remain adaptable and function effectively across different situations.^34^ In the present study, the moderate level of resilience observed among elderly participants suggests a somewhat limited ability to adjust to adverse circumstances, manage stressful events, and navigate the challenges associated with aging. It is advisable to train resilience-building programs at comprehensive health centers and development of community-based support networks to assist older adults in enhancing their coping abilities.

In this study, the total score for health-related quality of life was moderate, with older individuals exhibiting higher mental health compared to their physical health. The findings of Rahimi et al.'s ^35^ study were consistent with our results. However, Lou et al. ^36^ found that the physical health status of the elderly was better than their mental healts. The differences in findings could be due to the diversity in research techniques and the tools used to assess health status. Our study was conducted cross-sectionally, with the health status of the elderly assessed using the SF-12 questionnaire. In contrast, Lou et al. carried out a longitudinal study spanning 21 years, from 1994 to 2014. The evaluation of physical health took into account chronic illnesses, functional limitations, and challenges in carrying out daily tasks, whereas mental health was measured through the Center for Epidemiological Studies-Depression (CES-D) scale. The application of these various assessment techniques led to the varied results observed. Another possible explanation for the differing results could be related to variations in health system policies, and historical events in the societies under study. As the population of older adults continues to grow, it is advised that health care professionals perform screenings to recognize, refer, and initiate early intervention for the physical and mental health issues faced by the elderly. Additionally, encouraging healthy lifestyle practices before reaching old age can aid in minimizing the impact of diseases and disabilities during this phase of life.

The findings of the research indicated a notable link between experiencing abuse and a drop in mental health scores among the elderly. Cuevas et al. ^21^ also reported results that align with this study. While the natural aging process can heighten vulnerability to issues like depression, anxiety, and loneliness, undergoing elder abuse constitutes a traumatic experience that can result in extended psychological suffering.^33^ This highlights the necessity of psychological interventions for abused elderly individuals, suggesting the enhancement of psychological services in comprehensive health centers.

According to the study results, elderly individuals who have been abused tend to have lower resilience scores. In essence, resilience serves as protective factor against elder abuse; however, this association may be attributed to reverse causality and lower resilience predisposes to an increased risk of abuse. The results of Yan et al.'s ^37^ research align with the findings of this study. However, a study that conducted by Cuevas et al. ^21^ found a weak correlation between elder abuse and resilience, suggesting that higher levels of resilience do not necessarily lead to fewer instances of abuse. Resilience, defined as the capacity to navigate life’s stresses and challenges, enables older adults with higher resilience levels to cope more effectively with difficult situations. Moreover, it may support them in managing the social and psychological consequences associated with elder abuse.^38^

The study’s findings revealed that participants who had not experienced elder abuse had significantly better health outcomes across all dimensions compared to those who had. This difference was particularly noticeable in their mental health status and aligns with the findings of Cuevas et al.^21^ As people age, they often face issues like depression, anxiety, and loneliness, but elder abuse represents a serious life stress that can lead to profound and lasting psychological harm.^38^ In this study, psychological abuse was found to be the most common type of mistreatment, which can negatively influence the quality of life and lead to a deterioration of mental health in older adults.

The study indicated that older adults with higher resilience scores generally exhibited better mental health, although no significant association was found between resilience and physical health. This finding suggests that resilience may act as a protective factor against mental health challenges associated with elder abuse and aligns with the results reported by Upasen et al.^39^ As a result, efforts focused on boosting resilience—like building self-esteem, offering essential resources, and encouraging participation in social and spiritual activities—could be helpful in aiding and enhancing the mental health of elder people.

This is important to note that the generalizability of our findings is limited, as the sample consisted of older adults attending comprehensive health centers in Sanandaj and was recruited through consecutive convenience sampling, Therefore, caution is warranted when applying these results to other populations or settings, and Implementing the proposed recommendations in such settings may also face practical challenges, including resource constraints and staff training needs. Furthermore, the questionnaire utilized to assess elder abuse only focused on economic, emotional, and physical dimensions, neglecting other potential aspects of elder abuse. Consequently, it is advisable for future studies to incorporate a population-based approach using detailed questionnaires that cover all aspects of elder abuse.

## Conclusion

The research found that a significant number of elderly individuals had faced various types of abuse, with psychological abuse being the most common. Instances of abuse were more frequent among people residing in rural regions and among those with less educational attainment. Additionally, participants who had been subjected to abuse demonstrated lower resilience and poorer mental health outcomes. This study emphasizes the prevalence of elder abuse and its detrimental impact on the mental health of senior individuals. However, this is important to note that the study focused only on elderly individuals attending comprehensive health centers, excluding those residing in care facilities or those who did not seek care at these centers. This limitation restricts the generalizability of the findings. Furthermore, the questionnaire utilized to assess elder abuse only focused on economic, emotional, and physical dimensions, neglecting other potential aspects of elder abuse. Consequently, it is advisable for future studies to incorporate a population-based approach using detailed questionnaires that cover all aspects of elder abuse. 

**Acknowledgments: **This article is the result of a Master's thesis in Midwifery, entitled "Investigation the Extent of Elder Abuse and its Relationship with the Resilience and Health of the Elderly who referred to Comprehensive Health Centers in Sanandaj, 2022”, which was approved by the Ethics Committee of Kurdistan University of Medical Sciences, Sanandaj, Iran with the IR.MUK.REC.1401.161. The authors would like to thank all participants who contributed to this study. 

**Declaration of AI: **The authors used ChatGPT (OpenAI) solely for language editing and improving the readability of the manuscript. All scientific content, data analysis, interpretations, and conclusions were developed and verified by the authors, who take full responsibility for the content of this article Authors’ Contribution: Leila Hasheminasab and DR Jamal Seidi supervised the study and oversaw the study design, implementation, and final reporting. Roya Asadi, as an MSc student in Midwifery, contributed to proposal writing, study implementation, and preparation of the final report. Serveh Parang contributed to the study design, implementation, and preparation of the final report as an advisor. Sargol Shafiei contributed to the study design, implementation, and preparation of the final report. DR Kamyar Mansouri performed the statistical analysis and data interpretation. All authors reviewed and approved the final manuscript. 
